# Fear of falling and depression in older adults: The mediating role of attitudes toward aging and social networks

**DOI:** 10.1371/journal.pone.0324415

**Published:** 2025-11-13

**Authors:** Dan Zhang, Ziqing Qi, Lulu Wu, Yali Mao, Jia Wang, Yue Zhang, Ruting Wang, Annuo Liu

**Affiliations:** School of Nursing, Anhui Medical University, Hefei, Anhui Province, China; Tokyo Metropolitan Institute of Geriatrics and Gerontology, JAPAN

## Abstract

**Objective:**

Falls represent a common injury among older adults, and fear of falling is a prevalent psychological stressor in this population. This study aims to investigate the relationship between fear of falling and depression in older adults, as well as the chained mediating effects of attitudes toward aging and social networks.

**Methods:**

Using stratified cluster sampling, 1,158 adults aged 60 and older were surveyed between July and August 2022. Instruments included the Modified Fall Effectiveness Scale (MFES), Geriatric Depression Scale-15 (GDS-15), Attitudes Toward Aging Questionnaire (AAQ), and Local Social Network Scale-6 (LSNS-6). Correlation analysis and mediation effect testing were conducted using SPSS 26.0 and Stata 18.0.

**Results:**

Fear of falling was positively correlated with depression (β = 0.36, *p* < 0.001). Attitudes toward aging (Effect = 0.13, 95% CI (0.09, 0.17)) and social networks (Effect = 0.09, 95% CI (0.05, 0.13)) served not only as independent mediators between fear of falling and depression but also as chain mediators (Effect = 0.02, 95% CI (0.01, 0.03)).

**Conclusion:**

Fear of falling is associated with depression in older adults through aging attitudes and social networks. Healthcare providers should prioritize addressing older adults’ fear of falling and develop strategies based on this pathway to reduce the risk of depression in older adults.

## 1. Introduction

As populations age, the physical and mental health of older adults has received increasing attention. Depression is among the most prevalent mental health conditions in later life, with an estimated global prevalence of 35.1% [1]. It is associated with adverse health outcomes and marked declines in social functioning and quality of life [2,3]. The Notice on Comprehensively Strengthening Elderly Health Services issued by the National Health Commission emphasizes the importance of addressing mental health in older adults, calling for routine mental health assessments and follow-up management for common mental disorders and psychological/behavioral problems such as depression and anxiety. Prioritizing mental health in later life is essential to advancing healthy aging.

Falls are one of the most common injuries faced by older adults and have become the leading cause of injury-related deaths among those aged 65 and older [4]. According to Cognitive Stress Theory [5], an individual’s cognitive assessment of stress influences their emotional responses and coping strategies. As individuals age, they perceive a decline in their ability to control their environment and maintain independent living. This diminished confidence in their physical capabilities, coupled with an awareness of the serious consequences of falls, leads to a fear of falling. This fear becomes a major source of stress in later life. Research indicates that this fear of falling exists among both elderly individuals who have experienced falls and those who have not [6]. A significant association has been found between fear of falling and depression in older adults [7,8].

Attitudes toward aging refer to individuals’ beliefs, perceptions, and evaluations of their own aging and later life [9]. As a stressor, fear of falling is significantly related to attitudes toward aging [10]. Hobfoll’s Conservation of Resources Theory [11] posits that individuals possess a tendency to conserve, protect, and acquire resources; potential or actual resource loss triggers tension and stress in individuals. As individuals age, older adults simultaneously experience both the acquisition and accumulation of personal resources, as well as resource loss and functional decline. Research indicates that a positive attitude toward aging helps older adults cope with life stressors and maintain physical functioning [12,13], whereas a negative attitude toward aging accelerates cognitive decline and increases the risk of anxiety and depression [14,15].

A social network refers to the collection of social connections and relationships formed between individuals or organizations through social interactions [16]. The Theory of Planned Behavior (TPB) [17] posits that an individual’s behavioral intention is jointly determined by their attitude, subjective norm, and perceived behavioral control. Fear of falling reflects older adults’ diminished sense of self-control, potentially leading them to avoid social activities and resulting in a shrinking or absence of social networks. Multiple studies indicate that a lack of social networks can lead to depression, while supportive social networks help buffer individuals from the harmful effects of negative events and stressors [18,19]. Furthermore, attitudes toward aging are closely related to social networks. Research has found [20] that individuals’ attitudes toward aging influence their social networks and social support, which in turn impacts their quality of life.

Cognitive Behavioural Theory (CBT) posits that individuals’ thought patterns and cognitive schemas shape their emotions and behaviors, emphasizing the reciprocal interplay among cognition, emotion, and behavior [21]. With advancing age and declining physical function, older adults may construe falling as an unavoidable negative event in the aging process, thereby developing fear of falling as a maladaptive cognitive schema. As a dysfunctional cognitive pattern, fear of falling may foster negative attitudes toward aging and precipitate maladaptive behavioral responses. Older adults may adopt “activity avoidance” as a coping strategy to reduce their perceived risk of falling. However, activity avoidance can further lead to physical decline, diminished social roles, and shrinking social support networks, thereby exacerbating depressive symptoms and creating a vicious cycle. Furthermore, Social Emotional Selection Theory [22] posits that older adults increasingly value emotionally meaningful social relationships with advancing age. Thus, a positive attitude toward aging may strengthen motivation to maintain social networks, while a negative attitude may lead to social withdrawal. This provides theoretical support for the mediating role of aging attitudes in social network maintenance.

**Fig 1:**
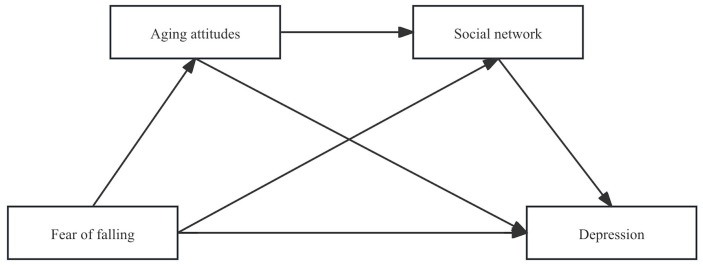
Chain mediation hypothesis model. Note: The predictor variable is fear of falling (measured by the Modified Fall Efficacy Scale with reverse scoring; higher scores indicate greater fear of falling).

Based on the above findings, fear of falling, attitudes toward aging, and social networks are each associated with depression in older adults and may be interrelated. However, research on their joint effects remains limited, and most studies emphasize single determinants rather than providing a comprehensive, multilevel account of pathways to late-life depression. Accordingly, this study examines the association between fear of falling (independent variable) and depression, specifying attitudes toward aging and social networks as mediators. We further investigate the mechanisms through which fear of falling may influence depression in older adults, with the aim of providing a theoretical basis for intervention development in geriatric depression. The research hypotheses are listed below, and the conceptual model is shown in Fig 1.

H1: Fear of falling is positively correlated with depression.
H2: Attitudes toward aging mediate the relationship between fear of falling and depression.
H3: Social networks mediate the relationship between fear of falling and depression.
H4: Attitudes toward aging and social networks exert a chain mediating effect between fear of falling and depression.

## 2. Subjects and methods

### 2.1. Study population

We used a stratified cluster sampling design in Yaohai District, Hefei, China, from July to August 2022. Strata were defined by urban and rural areas. From each stratum, two communities were selected by simple random sampling; within each community, two residential neighborhoods or village groups were randomly chosen as survey sites. Individuals aged 60 years and older were invited to participate. Inclusion criteria: (1) Age ≥ 60 years; (2) Clear consciousness and ability to complete the questionnaire; (3) Voluntary participation in the study and signed informed consent. Exclusion criteria: (1) History of psychiatric disorders or cognitive impairment; (2) History of severe cardiovascular or cerebrovascular disease; (3) Severe impairment in speech, vision, or hearing. This study was approved by the Ethics Committee of Anhui Medical University (Approval No.: 81220209).

### 2.2. Research instruments

#### 2.2.1. General information questionnaire.

A self-designed general information questionnaire was used to collect participants’ demographic characteristics. Variables included sex, age, current marital status, type of residence, educational attainment and living status.

#### 2.2.2. Modified Fall Efficacy Scale (MFES).

The MFES was developed by Hill et al. [23] as a further revision of the Fall Efficacy Scale (FES). It was adapted into Chinese by domestic scholars including Hao Yanping et al. [24] to assess older adults’ confidence in avoiding falls during specific activities. This measure evaluates participants’ fall efficacy, thereby reflecting the presence and severity of fall fear. The scale includes 14 items, with 9 covering indoor activities and 5 covering outdoor activities. Each item is rated on an 11-point scale from 0 (no confidence) to 10 (complete confidence). The final score is the mean of all items; lower scores reflect lower fall-related self-efficacy, corresponding to greater fear of falling. To align with the hypothesized direction, we reverse scored the MFES to create a “fear of falling” variable, such that higher values indicate greater fear. In this study, Cronbach’s α was 0.962.

#### 2.2.3. Geriatric Depression Scale (GDS-15).

The Geriatric Depression Scale translated and revised by Mei Jinrong et al. [25] was adopted. This 15-item scale ranges from 0 to 15 points, with higher scores indicating more severe depressive symptoms. A score ≥8 indicates depressive symptoms in the subject. In this study, the Cronbach’s α coefficient for this scale was 0.754.

#### 2.2.4. Attitudes to ageing Questionnaire (AAQ).

The Attitudes to ageing Questionnaire revised by Huang Yifan and colleagues [26] was used. The instrument contains 24 items across three domains: Psychological Growth, Physical Change, and Psychosocial Loss. Items are rated on a 5-point Likert scale, yielding a total score from 24 to 120. Items in the Psychosocial Loss domain are reverse scored. Higher scores indicate more positive attitudes toward aging. In this study, Cronbach’s α was 0.843.

#### 2.2.5. Lubben Social Network Scale-6 (LSNS-6).

The Lubben Social Network Scale-6, developed by Lubben et al. [27], was used. The instrument comprises two domains, family network and friends network, with six items in total. Items are rated on a 6-point Likert scale, producing a total score from 0 to 30. Each subscale ranges from 0 to 15. Higher scores indicate stronger family or friends networks among older adults. In this study, Cronbach’s α was 0.776.

### 2.3. Data collection methods

Questionnaires were collected from July to August 2022 through door-to-door visits and on-site surveys at community activity centers. All interviewers received standardized training. After providing written informed consent, the purpose of the survey was explained to participants. After obtaining informed consent, participants were guided to complete the questionnaire, which was collected on-site. Any omitted items were confirmed and supplemented if necessary. A total of 1,158 questionnaires were distributed. After excluding 39 questionnaires with missing data relevant to this study, 1,117 valid questionnaires were obtained, yielding an effective response rate of 96.46%.

### 2.4. Statistical methods

Data were analyzed using SPSS 26.0 and Stata 18.0. Common method bias tests were conducted in SPSS 26.0 to examine relationships among variables, while chained mediation effects were tested in Stata 18.0.

## 3. Results

### 3.1. Common method bias test

The Harman single-factor test was employed to examine common method bias. An exploratory factor analysis was conducted simultaneously on all items across four variables: fall efficacy, attitudes toward aging, social networks, and depression. Results revealed 14 factors with eigenvalues exceeding 1, with the largest factor explaining 18.84% of variance (<40%). Thus, no severe common method bias was identified in this study.

### 3.2. Demographic characteristics of participants

A total of 1,117 participants were included in the analysis: 440 men (39.4%) and 677 women (60.6%). By age group, 576 (51.6%) were 60–69 years, 420 (37.6%) were 70–79 years, and 121 (10.8%) were ≥80 years. The prevalence of depression differed significantly by age group, educational attainment, and residence type (p < 0.05). Additional characteristics are presented in Table 1.

**Table 1:** Generalization of depression conditions in the elderly (n = 1117).

Variables	n(%)	Depression score ($\begin{array}{c} \overset{―}{x} \end{array}$±s)	*t/F* Value	*p* Value
Sex			−1.766	0.078
Male	440 (39.4)	3.62 ± 3.00		
Female	667 (60.6)	3.94 ± 2.90		
Age, years			6.320	0.002
60 ~ 69	575 (51.6)	3.76 ± 2.97		
70 ~ 79	420 (37.6)	3.64 ± 2.91		
≥80	121 (10.8)	4.69 ± 2.82		
Type of residence			−1.28	0.203
Urban	950 (85.0)	3.77 ± 3.01		
Rural	167 (15.0)	4.05 ± 2.54		
Current marital status			1.287	0.198
Without spouse	172 (15.4)	4.08 ± 2.87		
With spouse	945 (84.6)	3.77 ± 2.96		
Educational attainment			5.236	0.001
Primary school or below	857 (76.7)	4.00 ± 2.97		
Junior high school	147 (13.2)	3.03 ± 2.56		
Senior high school/secondary vocational	92 (8.2)	3.40 ± 3.02		
College/associate degree	21 (1.9)	3.81 ± 3.30		
Living status			7.268	<0.001
Living alone	123 (11.0)	4.52 ± 3.07		
Living with spouse	471 (42.2)	3.66 ± 2.88		
Living with children	228 (20.4)	4.44 ± 3.04		
Living with spouse, children	287 (25.7)	3.29 ± 2.82		
Living with others	8 (0.7)	3.00 ± 1.60		

**Notes:**

a. Depression scores are presented as mean ± SD; categorical variables as n (%).

b. Group comparisons: independent-samples t tests were used for binary variables (sex, type of residence, current marital status), and one-way ANOVA was used for variables with ≥3 categories (age, educational attainment, living status). The corresponding t or F statistics are reported; *p* values are two-sided with α = 0.05.

c. Variable coding (for subsequent analyses): sex (1 = male, 2 = female);type of residence(1 = urban, 2 = rural);current marital status(0 = with spouse, 1 = without spouse); age (1 = 60–69 years, 2 = 70–79 years, 3 = ≥80 years); educational attainment (1 = primary school or below, 2 = junior high school, 3 = senior high school/secondary vocational, 4 = college/associate degree); living status(1 = living alone, 2 = living with spouse, 3 = living with children, 4 = living with spouse and children, 5 = living with others).

### 3.3. Descriptive statistics and correlation analysis

Correlation analysis of all variables yielded the results shown in Table 2. Significant correlations were found among fear of falling, attitudes toward aging, social networks, and depression. The mean scores for fear of falling, attitudes toward aging, social networks, and depression were 0.69, 75.05, 12.28, and 3.82, respectively. Fear of falling was negatively correlated with attitudes toward aging and social networks (r = −0.31, *p* < 0.001; r = −0.25, *p* < 0.001), and positively correlated with depression (r = 0.35, *p* < 0.001). Attitudes toward aging were positively correlated with social networks (r = 0.20, *p* < 0.001) and negatively correlated with depression (r = −0.32, *p* < 0.001). Social networks were negatively correlated with depression (r = −0.31, *p* < 0.001).

**Table 2:** Descriptive statistics and correlation analysis of variables.

Variables	M ± SD	1	2	3
1. Fear of falling	0.69 ± 1.39			
2. Aging attitudes	75.05 ± 9.54	−0.31***		
3. Social network	12.28 ± 4.97	−0.25***	0.20***	
4. Depression	3.82 ± 2.95	0.35***	−0.32***	−0.31***

Note: ***p < 0.001.

### 3.4. Chain mediation effect test

Using fear of falling as the independent variable and depression as the dependent variable, we established a chained mediation model with aging attitudes and social networks as mediating variables, while incorporating significant variables from univariate analysis (age, educational attainment, and living status) as covariates. Given the hierarchical cluster sampling design, we accounted for cluster effects by treating “community” as the clustering level for cluster-robust inference. Cluster-robust standard errors (CRSE) and their corresponding 95% confidence intervals were reported for all key models.

Test results are presented in Table 3. Fear of falling was positively correlated with depression (β = 0.36, *p* < 0.001) and negatively correlated with aging attitudes (β = −0.31, *p* < 0.001) and social networks (β = −0.20, *p* = 0.004). When fear of falling, aging attitudes, and social networks were simultaneously included in the regression equation, all three variables showed significant associations with depression. Fear of falling positively correlated with depression (β = 0.25, *p* < 0.001), while attitudes toward aging (β = −0.20, *p* = 0.003) and social networks (β = −0.21, *p* = 0.001) negatively correlated with depression. Attitudes toward aging positively correlated with social networks (β = 0.13, *p* = 0.004).

**Table 3:** Regression analysis of variable relationships in chained mediation.

Outcome Variables	Predictive variables	*R*	*R* ^*2*^	*F*	*β*	*t*	*p*	Cluster-robust SE	95%CI	
									Lower	Upper
Depression	Fear of falling	0.37	0.14	43.95	0.36	15.58	<0.001	0.07	0.30	0.41
Aging attitudes	Fear of falling	0.32	0.10	31.17	−0.31	−10.28	<0.001	0.21	−0.38	−0.24
Social network		0.30	0.09	21.68						
	Fear of falling				−0.20	−4.18	0.004	0.15	−0.32	−0.09
	Aging attitudes				0.13	4.12	0.004	0.03	0.06	0.21
Depression		0.47	0.22	52.62						
	Fear of falling				0.25	15.95	<0.001	0.08	0.21	0.28
	Aging attitudes				−0.20	−4.54	0.003	0.04	−0.30	−0.10
	Social network				−0.21	−6.03	0.001	0.03	−0.29	−0.13

**Fig 2:**
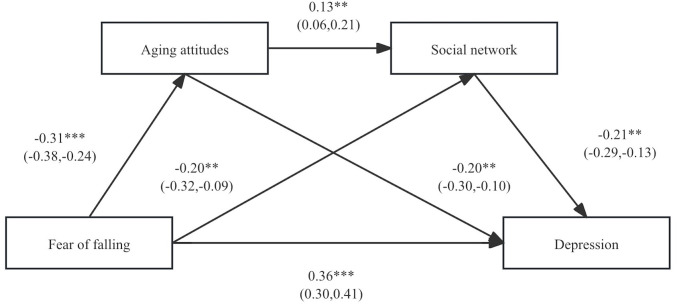
Chain mediation model of fear of falling predicting depression. Note: **p < 0.01; ***p < 0.001.

Further testing of mediating pathways revealed (see Table 4) that attitudes toward aging and social networks partially mediated the relationship between fear of falling and depression (Effect = 0.24, 95% CI (0.18, 0.29)), accounting for 31.58% of the total effect. The mediating effect of aging attitudes between fear of falling and depression was (Effect = 0.13, 95% CI (0.09, 0.17)), accounting for 17.11% of the total effect. The mediating effect of social networks between fear of falling and depression was (Effect = 0.09, 95% CI (0.05, 0.13)), accounting for 11.84% of the total effect. The chained mediating effect of aging attitudes and social networks between fear of falling and depression was (Effect = 0.02, 95% CI (0.01, 0.03)), accounting for 2.63% of the total effect. The chained mediation model diagram and path coefficients for each variable are shown in Fig 2.

**Table 4:** Mediating effect values and proportion of effect.

Type of effect	Pathway relationship	Effect value	cluster-robust SE	Relative mediation effect	*β*	*t*	*p*	Boot CI lower	Boot CI upper
Direct effect	Fear of falling→Depress-ion	0.52	0.06	68.42%	0.53	8.48	<0.001	0.39	0.62
Mediation effect	Fear of falling→Aging attitudes→Depression	0.13	0.05	17.11%	0.12	2.90	0.004	0.09	0.17
	Fear of falling→Social network→Depression	0.09	0.03	11.84%	0.10	2.94	0.003	0.05	0.13
	Fear of falling→Aging attitudes→Social network→Depression	0.02	0.02	2.63%	0.01	2.28	0.022	0.01	0.03
Total mediation effect		0.24	0.04	31.58%	0.25	3.07	0.002	0.18	0.29
Total effect		0.76	0.05	100%	0.75	12.52	<0.001	0.63	0.87

## 4. Discussion

### 4.1. Fear of falling is positively correlated with depression

Our findings show that fear of falling is positively associated with depressive symptoms (β = 0.36, *p* < 0.001). In other words, older adults reporting greater fear of falling were more likely to endorse depressive symptoms. Prior research suggests that advancing age, declines in physical function, and changes in social roles can weaken psychological defenses and emotion regulation, increasing vulnerability to mood problems, particularly depression [28]. When fear of falling develops, many older adults have difficulty using timely cognitive reappraisal to relieve negative affect. Persistent fear maintains heightened worry and tension, adds psychological burden, and can precipitate depressive symptoms.Therefore, fear of falling, as a specific negative psychological state in later life, has important implications for mental health. Healthcare providers should regularly assess the psychological well-being of older adults and use evidence-based interventions such as cognitive behavioral therapy to alleviate fear of falling and reduce the risk of depression.

### 4.2. Mediating role of attitudes toward aging

This study found a significant association between fear of falling and attitudes toward aging. Cross-cultural research indicates that sociocultural context strongly shapes these attitudes [29]. Western societies tend to emphasize personal independence and self-actualization, highlighting individual agency and perceived control in the aging process [30]. In contrast, in China, Confucian filial piety and collectivist values link attitudes toward aging more closely to family responsibilities and intergenerational support; these attitudes include both positive appraisals of longevity and experience and concerns about dependence and burden [31]. For many Chinese older adults, fear of falling may be construed as a visible marker of physical decline and instability, reinforcing negative self-perceptions of aging and deepening unfavorable attitudes toward the aging process. Empirical evidence shows that negative aging attitudes heighten stress responses to age-related stressors and increase depression risk [32], whereas positive attitudes facilitate coping, enhance life satisfaction, and reduce the likelihood of depression [33]. Consistent with this literature, our results indicate that attitudes toward aging mediate the relationship between fear of falling and depressive symptoms. The indirect effect accounted for 17.11% of the total effect and was larger than the other pathways examined. Encouraging and supporting more positive attitudes toward aging can strengthen self-confidence and self-efficacy in later life and may help lower the incidence of depression.

### 4.3. Mediating role of social networks

Mediation analysis showed that social networks mediate the association between fear of falling and depressive symptoms. With advancing age, fear of falling tends to increase. Heightened fear can lead to activity restriction and reluctance to participate in social activities, which over time reduces the size and quality of older adults’ networks. Evidence indicates that limited networks promote social isolation, contributing to frailty and depression [34]. In contrast, family and friend networks provide emotional and instrumental support that buffers the impact of negative life events [35], strengthens confidence in managing fall-related risks, and lowers vulnerability to depression. These findings highlight the need for society and families to promote social engagement among older adults by expanding opportunities for participation and encouraging sustained involvement in community and family activities.

### 4.4. Chain mediation of aging attitudes and social networks

The findings confirm the chain mediation effect of aging attitudes and social networks between fear of falling and depression. This aligns with Menkin et al.‘s [36] results, indicating that positive attitudes toward aging enable older adults to form more new friendships and develop richer social networks in later life. As a stressor, fear of falling can influence older adults’ perceptions of aging. Older adults with positive aging beliefs tend to counteract fear of falling by increasing physical and social activities [11]. Conversely, those holding negative aging beliefs often avoid stress by reducing activities, which diminishes the size of their social networks and subsequently impacts their emotional well-being. Therefore, it is crucial to help older individuals develop accurate perceptions of aging and encourage them to actively cultivate social networks.

### 4.5. Implications and limitations

This study elucidates the linkage between fear of falling and depression from dual perspectives of aging cognition and social support, thereby broadening the lens on risk factors for late-life mental health. Consistent with prior work, our findings underscore the salience of attitudes toward aging in coping with the aging process [37] and highlight the pivotal role of social networks in safeguarding psychological well-being among older adults. The results both echo international research on positive aging and mental health and foreground culturally specific risks in China, where family dependence and gaps in social support may heighten vulnerability. By incorporating a cross-cultural perspective, the study deepens understanding of aging attitudes and provides a theoretical basis for designing interventions that align with local sociocultural contexts. In addition, the findings emphasize the importance of cultivating positive attitudes toward aging and fostering supportive social networks to promote mental health and social well-being in later life, thereby offering theoretical support and practical avenues for advancing active aging strategies. The study also refines the application of psychological theories to the fear of falling–depression linkage and suggests new analytic directions for subsequent research.

The findings offer actionable guidance for policymakers, health care professionals, and community practitioners. For policymakers, aging services should integrate mental health and social support, including routine screening and intervention for fear of falling within medical and long-term care systems, environmental modifications that reduce fall risk, and public campaigns that promote positive views of aging and age-friendly communities. For health care professionals, interventions should extend beyond fall-prevention education to include psychological components that challenge negative aging beliefs and strengthen adaptive coping. For communities, expanding activity offerings and opportunities for social participation can enhance social support systems, build positive aging attitudes and resilience, and reduce depression risk. Coordinated, multilevel efforts across sectors are needed to promote integrated improvements in physical, psychological, and social functioning among older adults.

This study has limitations. First, the cross-sectional design precludes inferences about temporal ordering or causality. Accordingly, the term “serial mediation” denotes an association pattern consistent with the data rather than a demonstrated causal pathway. Second, we did not conduct multidimensional analyses of each construct, such as distinguishing family and friend components of social networks, which limits the specificity of guidance for practice. Future studies should incorporate multidimensional **measures** to yield more precise estimates and intervention targets.
